# Intermittent Hypoxia Exacerbates Lipopolysaccharide‐Induced Neurobehavioral Abnormalities: The Role of Neuroinflammation and Synaptic‐Related Proteins

**DOI:** 10.1002/brb3.71653

**Published:** 2026-08-05

**Authors:** Yi‐Zhu Ding, Zong‐Fang Wang, Chen Dai, Yuan‐Zi Ye

**Affiliations:** ^1^ Department of Infectious Diseases The First Affiliated Hospital of Anhui Medical University Hefei Anhui People's Republic of China; ^2^ Department of Neurological Surgery Anhui No. 2 Provincial People's Hospital Hefei Anhui People's Republic of China; ^3^ Thoracic Surgery Department Anhui Chest Hospital, Hefei, Anhui, People’s Republic of China; ^4^ Department of Pathology The First Affiliated Hospital of Anhui Medical University Hefei Anhui People's Republic of China

**Keywords:** cognition, intermittent hypoxia, lipopolysaccharide, neuroinflammation, synaptic proteins

## Abstract

**Background:**

Sepsis is a common systemic inflammatory disease clinically observed in patients with severe trauma or infectious diseases. Lipopolysaccharide (LPS) serves as a classic tool for studying infection‐related neuroinflammation and behavioral changes. Research indicates that LPS can induce peripheral inflammation and neuroinflammation, leading to synaptic dysfunction and neurobehavioral abnormalities. Furthermore, studies suggest that stress can increase susceptibility to inflammation. Intermittent hypoxia, a core pathological feature of obstructive sleep apnea syndrome, induces low‐grade chronic inflammation in the central nervous system.

**Objective:**

This study investigates whether intermittent hypoxia exacerbates LPS‐induced cognitive impairment, depression, and anxiety‐like behaviors, and explores its potential mechanisms.

**Methods:**

Adult mice were treated with LPS alone or in combination with daily 8‐h intermittent hypoxia via a hypoxia chamber. Depression and anxiety‐like behavior were assessed using the tail suspension, forced swim, elevated plus maze, and open field tests. Spatial learning and memory were measured using the Morris water maze test. Pro‐inflammatory cytokines and levels of genes and proteins of synaptic‐related proteins were detected using enzyme‐linked immunosorbent assay, western blotting, and real‐time fluorescence‐based quantitative PCR, respectively.

**Results:**

LPS‐induced impaired cognitive function, increased depression‐like and anxiety‐like behaviors, elevated pro‐inflammatory cytokines, and decreased mRNA and protein levels of synapse‐related proteins, synaptosomal‐associated protein of 25 kDa, synaptotagmin‐1, synaptophysin, and postsynaptic density protein‐95 in adult male mice. Intermittent hypoxia exacerbated these adverse effects.

**Conclusion:**

Intermittent hypoxia exacerbates LPS‐induced neurobehavioral abnormalities, which are associated with neuroinflammation and alterations in synapse‐related proteins.

## Graphical abstract

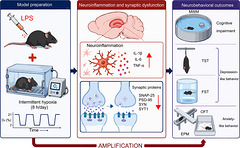

## 1 Introduction

Sepsis is a disease characterized by a systemic inflammatory response triggered by infection, leading to dysregulation of the body's immune system. It can cause multisystemic damage affecting the respiratory, cardiovascular, and nervous systems. About 11 million people worldwide die annually from sepsis‐related infections, making it a major public health crisis threatening lives and health (Goh et al. 2024). The central nervous system is particularly vulnerable to sepsis‐induced inflammation (Mazeraud et al. 2020; Iwashyna et al. 2010; Annane and Sharshar 2015; Basu et al. 2025). The mechanisms underlying these adverse neurological effects may involve neuroinflammation, disruption of the blood–brain barrier (BBB), glial cell activation, neuronal injury, and synaptic dysfunction. Crucially, the primary drivers of sepsis‐associated encephalopathy development are imbalances in the brain microenvironment and neuropathology, in which uncontrolled inflammatory responses and immune dysregulation play pivotal roles.

Neuroinflammation is an important pathogenic mechanism in psychiatric and neurodegenerative diseases, typically involving glial cell activation and the release of inflammatory mediators (Ding et al. 2022). During sepsis, inflammatory signals from the periphery can cross the BBB to reach multiple brain regions, including the hippocampus and cortex. Lipopolysaccharide (LPS) is a potent Toll‐like receptor 4 (TLR4) agonist, known to mimic pathogenic stimuli in inflammation and post‐inflammatory cognitive impairment (Lin et al. 2021). Peripheral LPS administration triggers activation of microglia and astrocytes, increased endoplasmic reticulum stress in the brain, and release of NF‐κB‐mediated cytokines (Yi et al. 2025; Tang et al. 2021), processes reflecting key features of neuroinflammation in neuropsychiatric disorders. Specifically, there are structural and functional alterations in the BBB as early as 4 hours after systemic inflammation, allowing peripheral immune cells and inflammatory factors to enter the central nervous system through the damaged BBB. These infiltrated cytokines can directly exert neurotoxicity and activate glial cells (Andonegui et al. 2018; Xiao et al. 2025). Overactivated microglia can release large amounts of reactive oxygen species and pro‐inflammatory cytokines such as tumor necrosis factor‐α (TNF‐α), interleukin‐6 (IL‐6), and interleukin‐1β (IL‐1) (Yan et al. 2022); activated astrocytes can produce excessive chemokine ligands (CXCL) and chemokines (CCL), and CCL11 secreted by astrocytes can further activate microglia, increasing the release of inflammatory factors (Farina et al. 2007). An increasing number of studies indicate that cytotoxic products and released inflammatory mediators induced by neuroinflammation can impair neuronal function and disrupt synaptic plasticity by altering synaptic proteins (Rao et al. 2012; Lima Giacobbo et al. 2019). SNAP‐25 (synaptosomal‐associated protein of 25 kDa), SYT1 (synaptotagmin‐1), postsynaptic density protein (PSD‐95), and synaptophysin (SYN) are common proteins which reflect synapse‐related functions. SNAP‐25, SYT1, and SYN are located on the presynaptic membrane, while PSD‐95 is located on the postsynaptic membrane. SNAP‐25 is a membrane‐associated protein belonging to the SNARE complex, primarily localized to the presynaptic membrane of neurons. It mediates the fusion of synaptic vesicles with the cell membrane, thereby facilitating neurotransmitter release. In addition to this classical function, SNAP‐25 also plays a role at the postsynaptic site, participating in receptor transport, dendritic spine morphogenesis, and synaptic plasticity (Fernström et al. 2018). Research indicates that SNAP‐25 maintains normal NMDAR levels, ensuring the normal induction of long‐term potentiation (LTP). When SNAP‐25 is knocked down in CA1 pyramidal cells in acute slices, the number of NMDARs (GluN1 subunit) on the dendritic surface is decreased to half, leading to impaired LTP (Jurado et al. 2013). Syt1 is a synaptic vesicle membrane–integrating protein primarily localized in the cytoplasm, containing C2 domains capable of binding calcium ions and phospholipids. It functions not only as a Ca^2^^+^ sensor but also as a multifunctional protein involved in synaptic vesicle fusion and endocytosis, and in various neurodevelopmental and transmission processes such as axonal growth (Greif et al. 2013). PSD‐95 and SYN are structural marker proteins for synaptic function and are also key proteins for maintaining LTP. Studies have demonstrated decreased levels of SYN and PSD‐95 proteins in the hippocampus and cerebellum of a valproic acid–induced mouse model of autism‐like behavior (Ling et al. 2023), and similarly reduced synaptic protein levels of SYN and PSD‐95 in mouse models of cognitive decline such as Alzheimer's disease (Ali et al. 2023). This evidence indicates that synaptic structure and function play crucial roles in cognitive function, depression, and anxiety. Furthermore, studies demonstrate that LPS exposure reduces PSD‐95 and SYN levels in the hippocampus of mice (Liang et al. 2023). Collectively, neuroinflammation and impaired synaptic function may be the pathological mechanisms underlying LPS‐induced emotional alterations and cognitive deficits in mice.

The various adverse stressors have been shown to increase susceptibility to inflammation and induce stronger neuroimmune responses, thereby causing neuropathological changes and cognitive alterations in the brain. Studies indicate that hyperglycemia exacerbates systemic inflammation and neuroinflammation in septic rats, leading to increased BBB permeability and cognitive deficits (Yao et al. 2024). Social defeat stress also exacerbates LPS‐induced anxiety‐like behaviors and cognitive impairments in mice via neuroinflammation (Ajayi et al. 2022). Obstructive sleep apnea (OSA) causing apnea and hypoventilation symptoms, is also considered a chronic low‐grade systemic inflammatory disease due to its intermittent hypoxia (IH) component (Kiernan et al. 2016). A growing body of research indicates that IH causes damage to brain structure and function, along with adverse behavioral changes such as mood alterations and cognitive decline (Tang et al. 2023; Miyo et al. 2025). IH itself, as a chronic stressor, can exacerbate LPS‐induced immune responses in the central nervous system by altering the brain microenvironment (Zhou et al. 2017). Notably, sepsis and noninfectious respiratory diseases co‐occur in patients, such as OSA and chronic obstructive pulmonary disease (COPD) (Liu et al. 2024). Therefore, there are significant clinical implications for investigating the potential effects and mechanisms of IH on LPS‐induced anxiety‐ and depression‐like behaviors, and cognitive impairments.

In this study, we used adult male C57BL/6J mice exposed to LPS to establish a sepsis model, and exposed them to an IH environment to establish an OSA model. The aim was to explore whether IH could exacerbate the increased anxiety‐ and depression‐like behaviors and cognitive dysfunction induced by LPS; if so, whether the underlying mechanisms involved neuroinflammation and impaired synaptic‐associated proteins in the hippocampus.

## 2 Materials and Methods

### 2.1 Animals

All mice were purchased from the Beijing Vital River Laboratory Animal Technology Co., Ltd (Shanghai, China) and maintained under specific pathogen‐free conditions with 21°C–23°C and 50%–60% relative humidity under a 12‐h light/dark cycle. Following 2‐weeks adaptation phase before the start of the experiment, all animals had ad libitum access to water and food. All animal model and behavioral procedures were conducted in accordance with the ARRIVE guidelines and approval was obtained from the Animal Ethics Committee of Anhui Medical University.

### 2.2 Experimental Design and Animal Model

**FIGURE 1:**
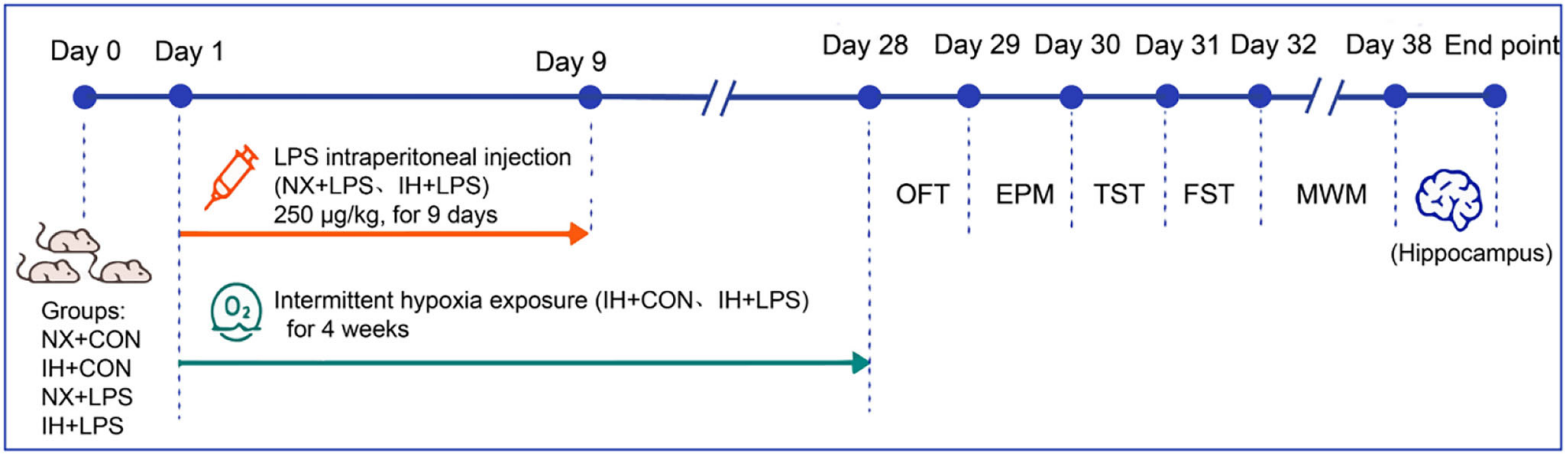
Schematic overview of the experimental design and timeline. EPM, elevated plus maze; FST, forced swimming test; MWM, Morris water maze; OFT, open field test; TST, tail suspension test.

These mice were randomly assigned to normoxia + control (NX + CON), NX + LPS, and IH + LPS groups. Mice assigned to the NX + LPS and IH + LPS groups were administered LPS (250 µg/kg, *Escherichia coli* 0127: B8, Sigma, USA.) via intraperitoneal injection for 9 consecutive days. Mice in the IH + CON and IH + LPS groups were exposed to hypoxia (Figure 1).

### 2.3 IH Treatment

The IH paradigm consisted of alternating cycles of NX (21% FiO_2_) and hypoxia (7% FiO_2_), delivered at a frequency of 20 cycles per hour for 8 h daily (09:00–17:00). Each hypoxia–reoxygenation cycle lasted 180 s and comprised four sequential phases: a reduction in oxygen concentration from 21% to 7% within 30 s, a hypoxic exposure period maintained at 7% FiO_2_ for 60 s, a reoxygenation phase returning to 21% FiO_2_ within 30 s, and a normoxic stabilization period maintained at 21% FiO_2_ for 60 s. Oxygen concentration was precisely controlled using a programmable, feedback‐regulated gas delivery system to ensure stable and reproducible FiO_2_ transitions throughout the exposure period (ProOx‐100, Shanghai, China). This IH protocol was continuously applied for 4 weeks to establish a chronic IH model.

### 2.4 Open Field Test

The open field test was used to assess anxiety‐like behavior. This test was conducted in a gray box measuring 50 × 50 × 40 cm^3^, illuminated at 120 lux. Each mouse was placed at the center of the arena and allowed to freely explore for 5 min. After each trial, the apparatus was wiped with 75% ethanol to eliminate olfactory cues and prevent interference with subsequent trials. Mouse activity within the arena was recorded using the smart software. Quantitative parameters, including total distance traveled, central time, central distance, and the number of times into the center area (defined as a 25 × 25 cm^2^ area in the center), were analyzed during the 5‐min observation period.

### 2.5 Elevated Plus Maze Test

The apparatus consists of a raised plus‐shaped maze positioned 80 cm above the ground, featuring four arms and a central platform. Each arm measures 6 cm in width and 30 cm in length, with the closed arms having 15 cm‐high walls, whereas the open arms have no walls. The central area measures 6 × 6 cm. At the start of the experiment, each mouse was placed in the central platform facing one of the open arms and allowed to explore freely for 6 min. All mouse movements were recorded using the smart software.

### 2.6 Tail Suspension Test

Each mouse was suspended upside down 35 cm above the ground by taping its tail. The entire 6‐min session was video‐recorded, and the duration of immobility during the last 4 min was quantified. An increase in immobility time is considered indicative of enhanced depression‐like behavior, reflecting a state of behavioral despair.

### 2.7 Forced Swimming Test

The apparatus consisted of a cylindrical container (9 cm in diameter, 28 cm in height) filled with water at a depth of 15 cm and maintained at 21°C–23°C. Each mouse was placed in a bucket filled with water for a total duration of 6 min, and the entire session was video‐recorded. Consistent with the tail suspension test, the last 4 min were analyzed to quantify immobility time. An increase in immobility time in the forced swim test is similarly interpreted as an indicator of heightened depression‐like behavior.

### 2.8 Morris Water Maze Test

The device consisted of a circular pool and a cylindrical escape platform. The quadrant containing the hidden platform was designated as the target quadrant. During the training phase, each mouse underwent four trials per day for 5 days. In each trial, the mouse was gently placed in the water and allowed to swim freely for 60 s. When a mouse successfully found the hidden platform, it was permitted to stay on it for 30 s. If the mouse did not locate the platform within 60 s, the experimenter gently guided it to the platform, where it was likewise allowed to rest for 30 s. Each mouse was introduced into the pool from different quadrants each day in a randomized order. A video tracking system was used to record all movements of the mice. On the 6th day, a probe test was conducted by removing the platform. The mouse was placed in the water from the opposite side of the target quadrant and allowed to swim freely for 60 s. The behavioral system records the movement time and distance of mice in the target quadrant to assess memory retention.

### 2.9 Brain Tissue Extraction

Following behavioral experiments, mice were euthanized using 1.5% sodium pentobarbital. Brain tissue was rapidly removed by dissecting the skull. The hippocampal tissue was gently isolated on ice and placed into 2 mL centrifuge tubes. These tubes were rapidly frozen in liquid nitrogen and subsequently transferred to a refrigerator maintained at −80°C for storage. The hippocampal tissue was reserved for subsequent protein and RNA extraction.

### 2.10 Enzyme‐Linked Immunosorbent Assays

The levels of IL‐1β, TNF‐α, and IL‐6 were detected using ELISA kits (JYM0531Mo, JYM0218Mo, JYM0012Mo; Jiyinmei, China) in hippocampal tissues from four groups of mice. Following the kit's operating instructions, the specific steps were as follows: Hippocampal tissues were removed and weighed, then minced. An appropriate amount of PBS was added for thorough homogenization, followed by centrifugation at 2500 rpm for 20 min. The supernatant was reserved for subsequent use. Standard wells (containing different concentrations of standards), blank wells (control wells without sample or enzyme‐labeled reagents, otherwise processed identically to other wells), and sample wells were prepared. The experimental process was carried out in sequence according to the steps in the manual. Finally, the absorbance (OD value) was measured using an ELISA reader at 450 nm.

### 2.11 Real‐Time Fluorescence‐Based Quantitative PCR (RT‐qPCR)

Total RNA was obtained from hippocampal tissues using TRIzol reagent (Life Technologies, USA). After RNA extraction, the concentration and purity of the samples were measured using the NanoDrop 2000 micro‐volume spectrophotometer to verify that they met the quality standards required for subsequent experiments. The extracted RNA was reverse transcribed into cDNA using the 5× Evo M‐MLV RT Master Mix (Accurate Biology, AG11706). The qPCR mixture contained forward and reverse primers, 2× SYBR Green Mix, cDNA, and RNase‐free water. The real‐time fluorescence quantitative PCR instrument (Thermo Fisher Scientific, USA) conducts the reaction. The relative gene expression levels were calculated using the 2^−ΔΔCt^ method, with β‐actin used as the internal reference gene. The primers used for qPCR are described in Table 1.

**TABLE 1:** RT‐PCR primers’ sequence details.

Gene	Amplicon size (bp)	Forward primer (5'→3')	Reverse primer (5'→3')
β‐actin	120	AGTGTGACGTTGACATCCGT	TGCTAGGAGCCAGAGCAGTA
SNAP‐25	94	AGCCTGCTCGTGTAGTGG	TCGATCTGGCGATTCTGT
SYT1	104	GTGACCTGCAAAGTGCTGAGAA	ACCAGCAGTAGGTACGTAGCGAAG
PSD95	72	CCCAGGATATGTGAACGGAA	CCTGAGTTACCCCTTTCCAA
SYN	124	GCCTACCTTCTCCACCCTTT	GCACTACCAACGTCACAGAC

### 2.12 Western Blotting

The hippocampal tissue is extracted and weighed. For every 10 mg of tissue, 100 µL of tissue lysis buffer is added (Beyotime, P0013B). The tissue is then homogenized on ice using a homogenizer. Subsequently, the homogenate is centrifuged at 12,000 rpm for 15 min at 4°C. The supernatant is collected. Protein concentration is determined using a BCA protein assay kit (Thermo Scientific, A55864). Based on the measured protein concentration, the required volume for loading is calculated. The extracted protein supernatant is mixed with 5× loading buffer at a ratio of 4:1 and incubated in a metal bath at 100°C for 10 min to fully denature the proteins. After denaturation, the samples are cooled on ice and directly loaded into the wells of an SDS‐PAGE gel. Electrophoresis is first performed at a constant voltage of 80 V until the bromophenol blue dye reaches the interface between the concentrating gel and separation gel, at which point the voltage is increased to 120 V. Electrophoresis continues until the dye front reaches the bottom of the gel. The entire electrophoresis process takes approximately 1.5 h. After electrophoresis, the SDS‐PAGE gel is transferred to a membrane transfer apparatus for blotting. The PVDF membrane must be activated in methanol before use. During the transfer, care should be taken to avoid air bubbles between the gel and the PVDF membrane. Membrane transfer is performed under constant current conditions, with transfer time adjusted based on the molecular weight of the target proteins. After transfer, the PVDF membrane is blocked in TBST solution containing 5% nonfat milk for 1 h on a shaker at room temperature. Primary antibodies are then diluted at the following ratios: PSD‐95 (1:1000, Abcam, ab238135), Syt1 (1:1000, Bioss, bs‐4172R), SNAP‐25 (1:1000, Bioss, bsm‐60238 M), SYN (1:1000, Bioss, bs‐8845R), GAPDH (1:5000, Zsbio, TA‐08). After blocking, the PVDF membrane is washed three times with TBST, each wash lasting 5 min. The membrane is then incubated with the diluted primary antibody solution overnight at 4°C. The following day, the membrane is washed thoroughly with TBST (three times, 10 min each). Horseradish peroxidase–conjugated secondary antibodies (Zsbio, ZB‐2305/ZB‐2301) are diluted at 1:20,000 in secondary antibody diluent, and the membrane is incubated with this solution on a shaker at room temperature for 1 h. Then, the membrane is washed again with TBST (three times, 10 min each). Protein bands on the PVDF membrane are detected using ECL working solution. Finally, protein band intensity is analyzed using Fiji (ImageJ) software.

### 2.13 Immunofluorescence Staining

Immunofluorescence staining was performed on brain tissue sections. The procedure consists of tissue preparation and staining. Briefly, the tissue preparation procedure involved anesthetizing mice with sodium pentobarbital (P3761, Sigma–Aldrich) and performing cardiac perfusion with PBS, followed by overnight fixation in 4% PFA (P0099, Beyotime) at 4°C. The brain tissue was then sequentially dehydrated in 20% and 30% sucrose solutions until complete sedimentation. Brain tissue was sectioned at a thickness of 20 µm using a freezing microtome (FS800A, RWD). For immunostaining, tissue sections were first washed three times with PBS for 3 min each, followed by treatment with 0.2% Triton X‐100 (T8787, Sigma–Aldrich) for 15 min. To reduce nonspecific background binding, the sections were incubated at room temperature in a blocking buffer containing 5% bovine serum albumin (A8010, Solarbio) for 1 h. Subsequently, these sections were incubated overnight at 4°C with primary antibodies, Iba1 (1:1000, ab283346, Abcam) and GFAP (1:1000, ab227641, Abcam). The next day, after washing three times with PBS, the sections were incubated with the corresponding fluorescently labeled secondary antibodies–goat anti‐rabbit (1:1000, A‐11008, Invitrogen) and rat IgG (1:1000, A‐21247, Invitrogen) for 1 h at room temperature in the dark. Cell nuclei were stained by incubating with DAPI solution for 10 min. Finally, images were captured using a confocal microscope (SP8, Leica).

### 2.14 Statistical Analysis

All results are presented as mean ± standard error (mean ± SEM). Statistical analysis was performed using GraphPad Prism 9.0 (GraphPad Software, Inc.). The Shapiro–Wilk test was applied to all data to assess normality. Based on this test, one‐way analysis of variance (ANOVA) followed by Tukey's post hoc test was used for multiple group comparisons. Two‐way ANOVA followed by Tukey's post hoc test was employed to compare the escape latency, swimming distance, and swimming speed during the learning phase of the Morris water maze test. *p*‐value < 0.05 was considered statistically significant.

## 3 Results

### 3.1 Effect of IH on Anxiety‐Like Behaviors Induced by LPS

**FIGURE 2:**
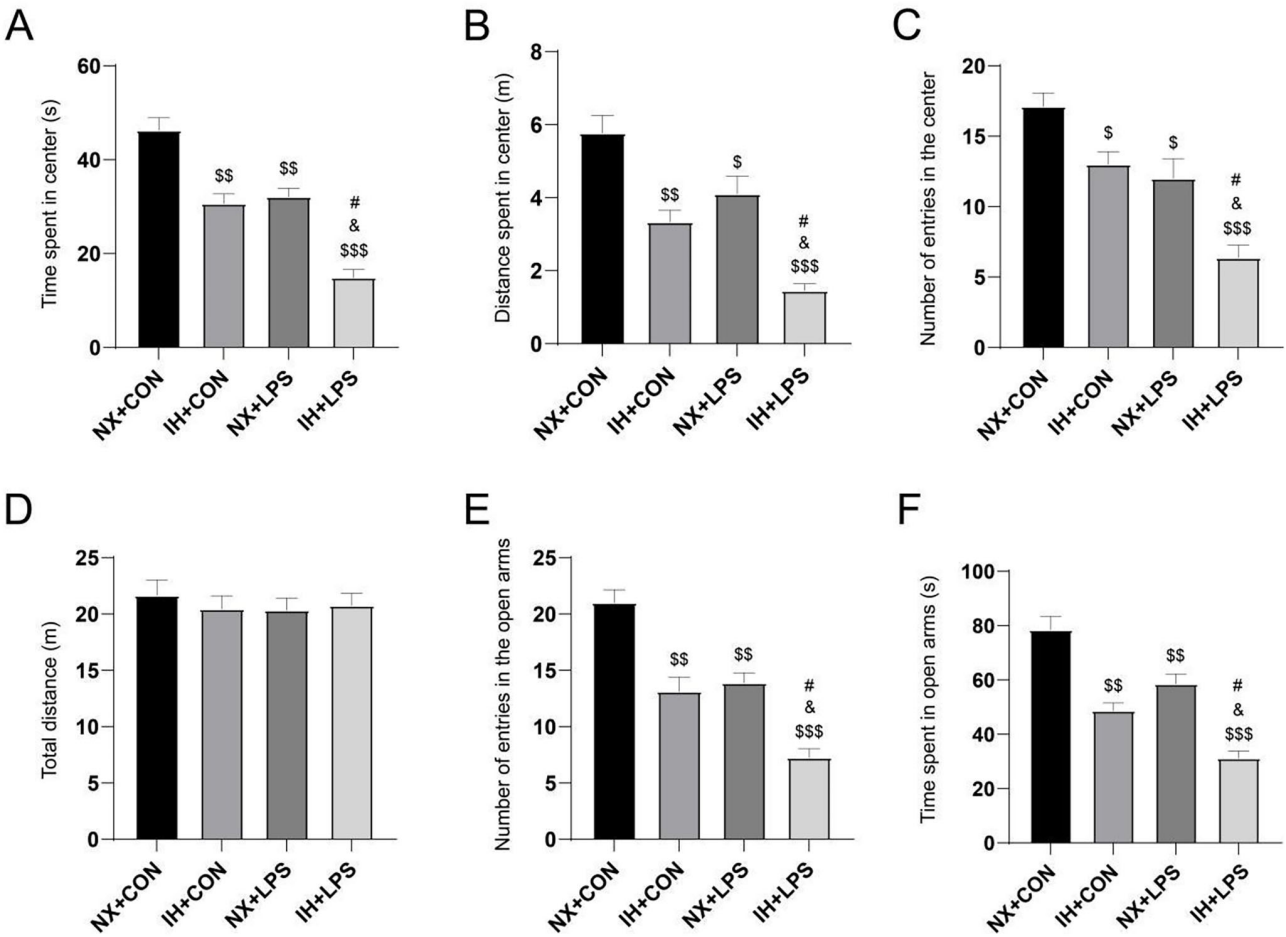
IH increased the anxiety‐like behavior induced by LPS (*n* = 8). (A) Time spent, (B) distance traveled in center region, (C) frequency of entering center area, and (D) the entire distance are displayed in open field test. (E) Frequency of entry to (F) the time spent in open arms are shown in elevated plus maze test. *^$^p* < 0.05, *^$$^p* < 0.01, and *^$$$^p* < 0.001 for intergroup comparison with NX + CON; *^&^p* < 0.05 for intergroup comparison with IH + CON group; *^#^p* < 0.05 for intergroup comparison with NX + LPS group.

The open field test showed statistically significant differences between the four experimental groups in terms of the number of entry in the central zone, the amount of time spent there, and the distance traveled (time: *F*_(3, 28)_ = 38.37; distance: *F*_(3, 28)_ = 21.21; number of entries: *F*_(3, 28)_ = 17.25) (Figure 2A–C). Follow‐up post hoc analysis revealed that animals challenged with LPS exhibited a marked decline in center—related behaviors—specifically, reduced time, distance, and entry frequency, compared to the NX + CON group. Moreover, mice in the IH + LPS group showed significantly lower values across these same parameters relative to both the IH + CON and NX + LPS groups. However, total locomotor activity, as indicated by the total distance traveled, did not differ significantly across the groups (Figure 2D).

In the elevated plus maze, the results revealed substantial group effects on both the number of admissions into open arms (*F*_(3, 28)_ = 28.29) and the quantity of time spent there (*F*_(3, 28)_ = 29.71) (Figure 2E,F). Post hoc comparisons showed that mice exposed to LPS spent significantly less time in the open arms and entered them less frequently than those in the NX + CON group. Furthermore, IH in combination with LPS resulted in a more pronounced reduction in these anxiety‐related measures compared to the NX + LPS group.

### 3.2 Effect of IH on Depression‐Like Behaviors Induced by LPS

**FIGURE 3:**
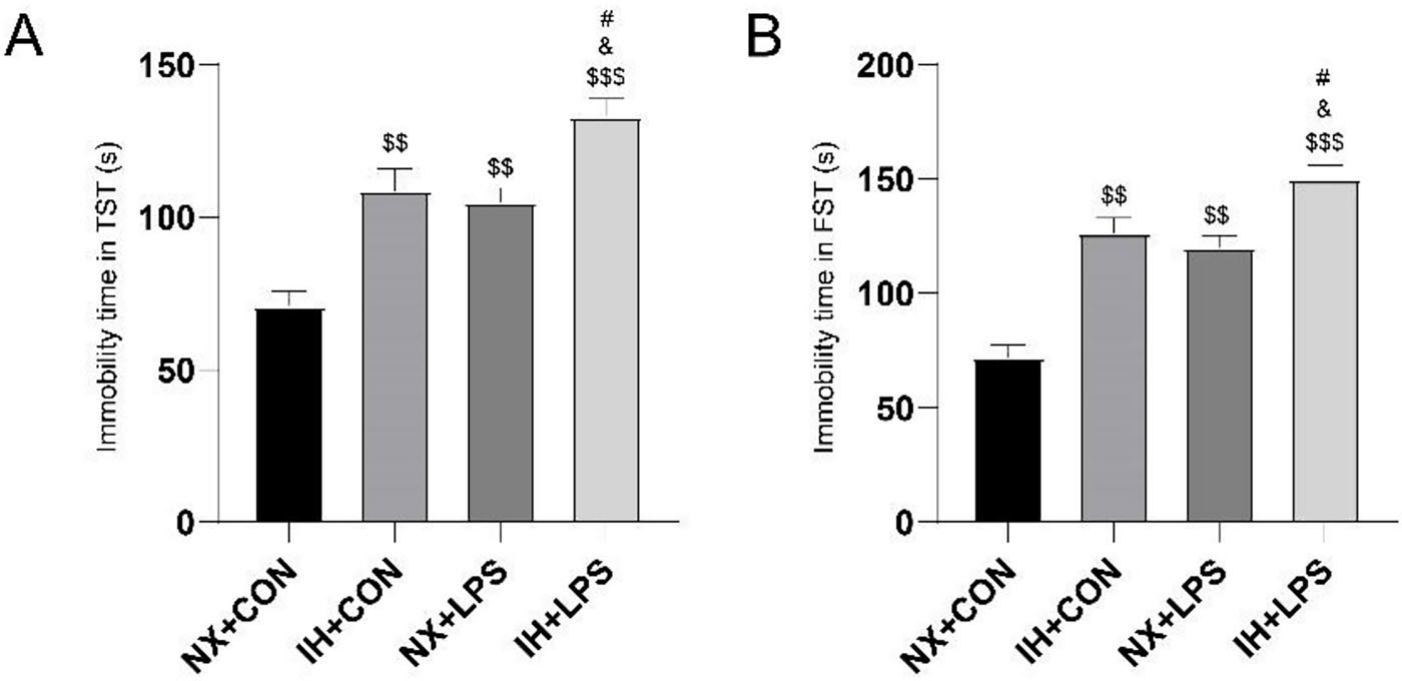
IH increased the depression‐like behavior induced by LPS (*n* = 8). (A) The immobility time is shown in tail suspension test. (B) The immobility time is shown in forced swimming test. *^$$^p* < 0.01 and *^$$$^p* < 0.001 show differences from NX + CON group; *^&^p* < 0.05 show differences from IH + CON group; *^#^p* < 0.05 show differences from NX + LPS group.

The effects of exposure to IH and LPS on depressive‐like behaviors were evaluated using tail suspension and forced swimming tests. The results of the tail suspension test showed that immobility time varied significantly across the four experimental groups (*F*_(3, 28)_ = 19.28) (Figure 3A). Post hoc analysis indicated that the NX + LPS group exhibited significantly higher immobility time compared to the NX + CON group, and the IH + LPS group had even longer immobility duration than the NX + LPS group. Similarly, the forced swimming test showed significant group differences in immobility time (*F*_(3, 28)_ = 30.79) (Figure 3B). After‐hoc comparisons revealed that IH exposure further increased immobility time in IH + LPS group compared to NX + LPS group.

### 3.3 Effect of IH on Learning and Memory Dysfunction Induced by LPS

#### 3.3.1 Learning Phase

**FIGURE 4:**
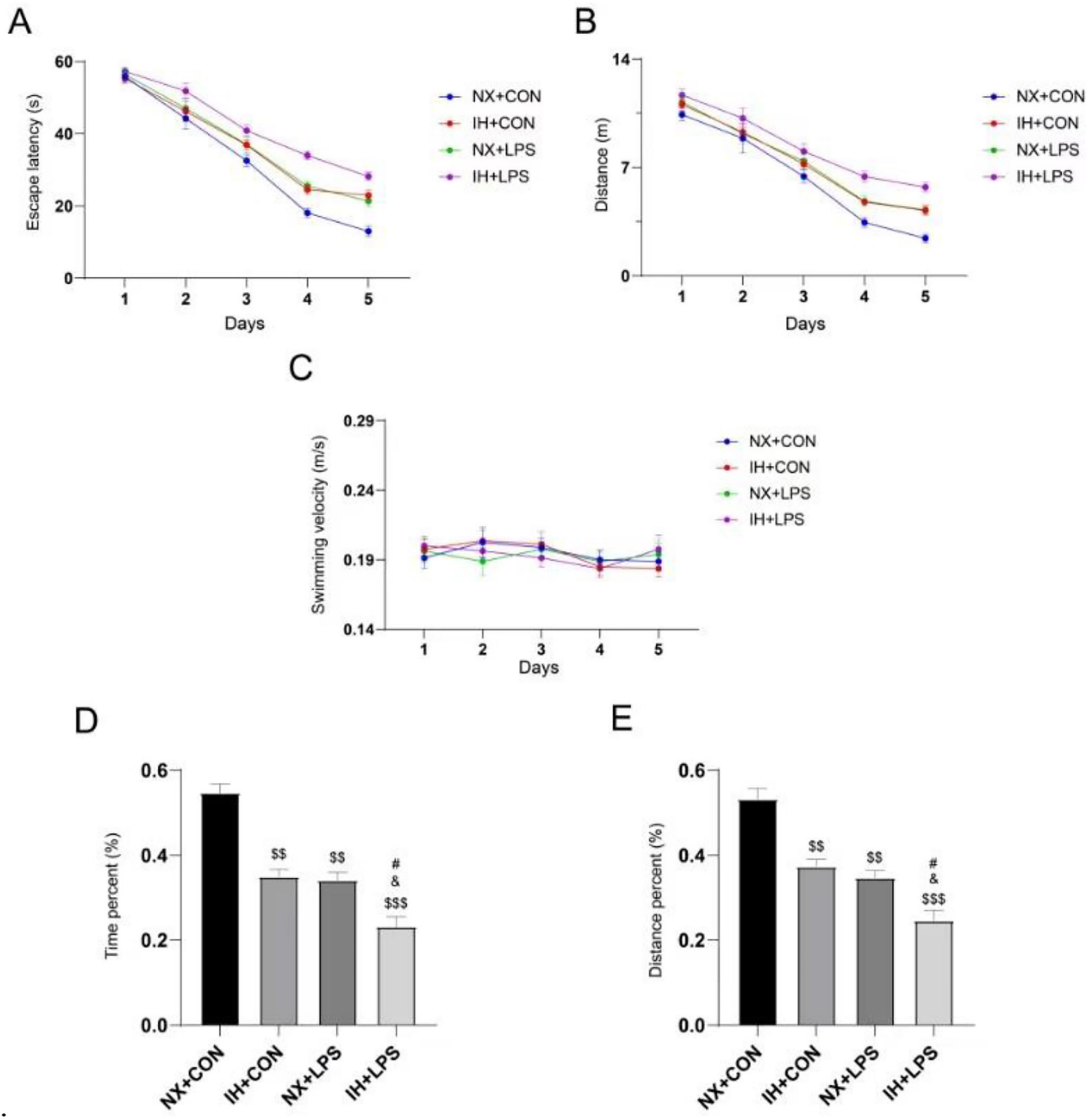
IH further impairs LPS‐induced deficits in spatial learning and memory abilities (*n* = 8). (A) Escape latency, (B) swimming distance, and (C) swimming velocity are presented during training phase. (D) Percentage of time and (E) distance in target quadrant are displayed in memory phase. *^$$^p* < 0.01 and *^$$$^p* < 0.001 denote significant differences relative to NX + CON group; *^&^p* < 0.05 signifies significant differences in comparison to IH + CON group; *^#^p* < 0.05 indicate differences relative to NX + LPS group.

During acquisition phase, results revealed significant main effects of treatment and training day on escape latency (treatment: *F*_(3, 28)_ = 22.01; training day: *F*_(4, 112)_ = 268.8) as well as swimming distance (treatment: *F*_(3, 28)_ = 15.31; training day: *F*_(4, 112)_ = 172.2) (Figure 4A,B). Notably, mice in IH + CON and NX + LPS groups exhibited prolonged escape times and increased swim paths compared to the NX + CON controls. Moreover, exposure to IH and LPS further exacerbated these impairments, as evidenced by longer escape latencies and greater swimming distances in IH + LPS group relative to NX + LPS group. However, there were no significant group differences in swimming speed (Figure 4C).

#### 3.3.2 Memory Phase

In the probe trial, spatial memory performance was assessed by quantifying the percent of time allocated and the distance traversed within the target quadrant. Analysis revealed marked differences among the groups for both metrics (time: *F*_(3, 28)_ = 45.46; distance: *F*_(3, 28)_ = 32.61) (Figure 4D,E). Significant reductions in both time and distance in target zone were observed in NX + LPS group relative to NX + CON group, indicating impaired memory retention. These deficits were further amplified in the IH + LPS group, which showed even poorer performance relative to the NX + LPS group.

### 3.4 Effects of IH on LPS‐Induced Glial Cell Activation and Pro‐Inflammatory Cytokine Elevation in the Hippocampus

**FIGURE 5:**
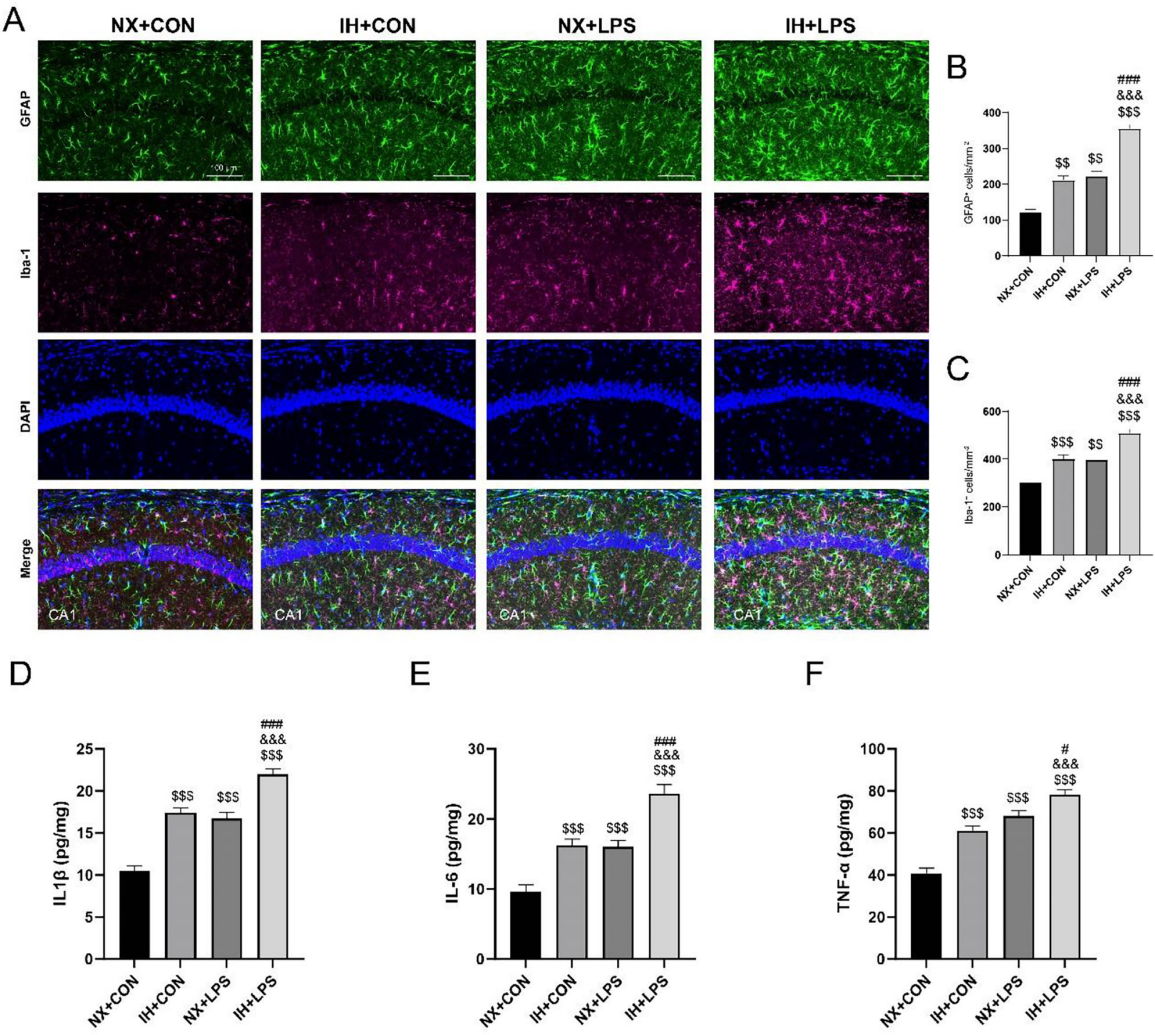
IH further increased the LPS‐induced glial cell activation and rise in the pro‐inflammatory cytokine. (A) Representative immunofluorescence images of GFAP^+^ astrocytes (green), Iba‐1^+^ microglia (magenta), and DAPI‐stained nuclei (blue) in the CA1 region of the hippocampus. Scale bar = 100 µm. Bar graphs illustrating the numbers of (B) GFAP^+^ astrocytes and (C) Iba‐1^+^ microglia in the CA1 region of each group (*n* = 6). Bar graphs of hippocampal (D) IL‐1β, (E) IL‐6, and (F) TNF‐α concentrations of each group (*n* = 8). *^$$^p* < 0.01 and *^$$$^p* < 0.001 show differences from NX + CON group; *^&&&^p* < 0.001 show differences from IH + CON group; *^#^p* < 0.05 and *^###^p* < 0.001 show differences from NX + LPS group.

We performed immunofluorescence staining to examine the alterations of astrocytes and microglia across the four experimental groups (Figure 5A). Significant differences were observed in the numbers of GFAP positive (*F*_(3, 20)_ = 75.25) and Iba‐1 positive (*F*_(3, 20)_ = 32.48) cells among the groups (Figure 5B,C) in the CA1 region of the hippocampus. Post hoc comparisons revealed that NX + LPS group significantly increased the counts of both GFAP positive and Iba‐1 positive cells compared to the controls indicating a neuroinflammatory response characterized by glial activation. Similarly, mice exposed to IH alone also displayed an elevation in the numbers of these cells. It is worth noting that the combination of IH and LPS treatment further increased the number of GFAP positive and Iba1 positive cells. Significant differences were observed in hippocampal concentrations of IL‐1β (*F*_(3, 28)_ = 57.01), IL‐6 (*F*_(3, 28)_ = 29.70), and TNF‐α (*F*_(3, 28)_ = 40.90) across the four groups (Figure 5D–F). Subsequent post hoc testing revealed elevated concentrations of these inflammatory cytokines in NX + LPS group relative to NX + CON controls. Moreover, IH exposure elevated their concentrations in IH + LPS group relative to NX + LPS group.

### 3.5 Effect of IH on LPS‐Induced Alterations in mRNA Levels of Synaptic‐Related Proteins

**FIGURE 6:**
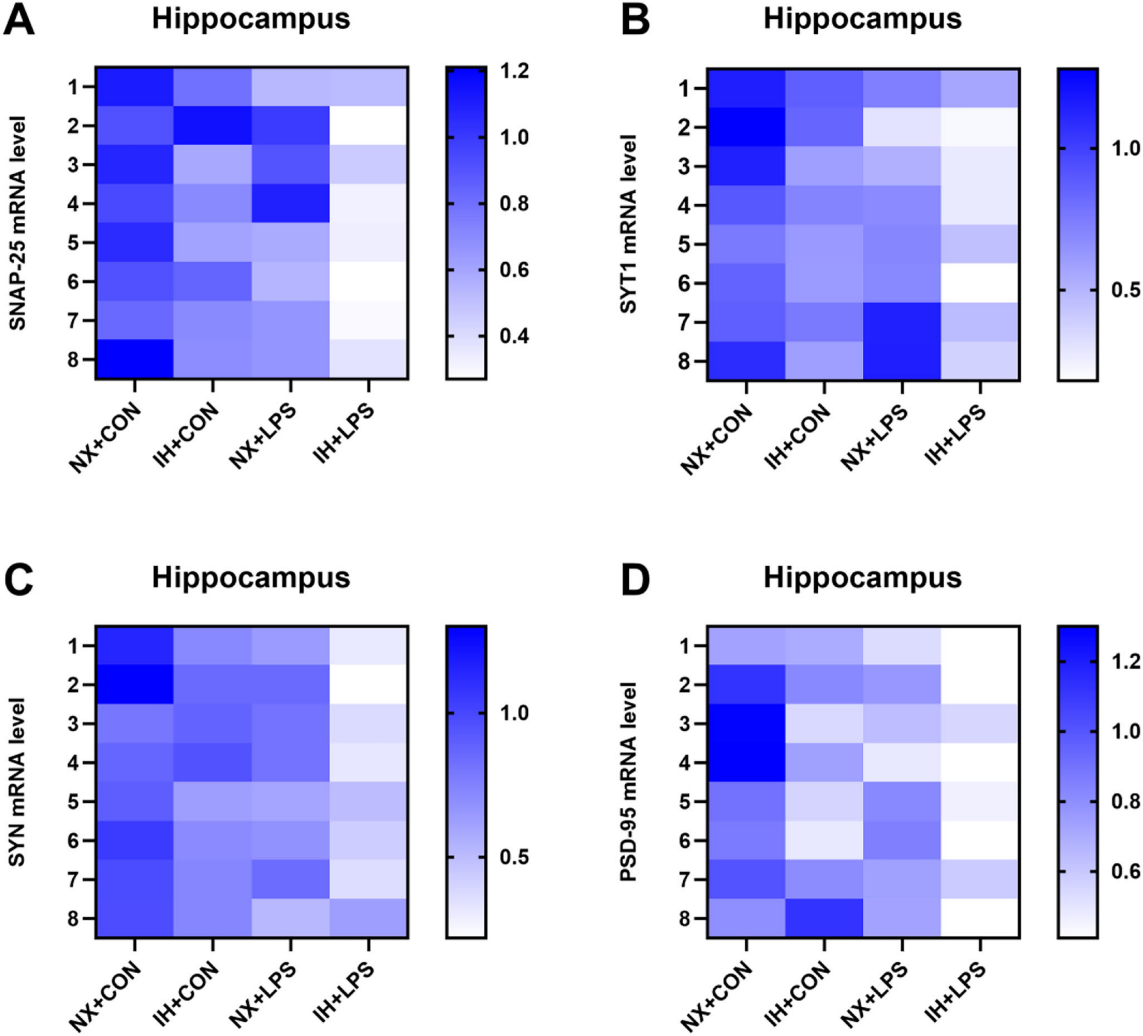
IH further reduced hippocampal mRNA levels of SNAP‐25, SYT1, SYN, and PSD‐95 in the NX + LPS group (*n* = 8). The heat map of hippocampal (A) SNAP‐25, (B) SYT1, (C) SYN, and (D) PSD‐95 are shown.

Results indicated significant differences in the mRNA levels of SNAP‐25 (*F*_(3, 28)_ = 22.06), SYT1 (*F*_(3, 28)_ = 15.68), SYN (*F*_(3, 28)_ = 29.30), and PSD‐95 (*F*_(3, 28)_ = 17.09) between the four groups (Figure 6A–D). The NX + LPS group exhibited significantly lower mRNA expression levels of SNAP‐25, SYT1, SYN, and PSD‐95 relative to NX + CON group. Furthermore, the transcripts of these synaptic markers were significantly downregulated in IH + LPS group relative to NX + LPS group.

### 3.6 Effect of IH on LPS‐Induced Changes in Protein Levels of Synaptic‐Related Proteins

**FIGURE 7:**
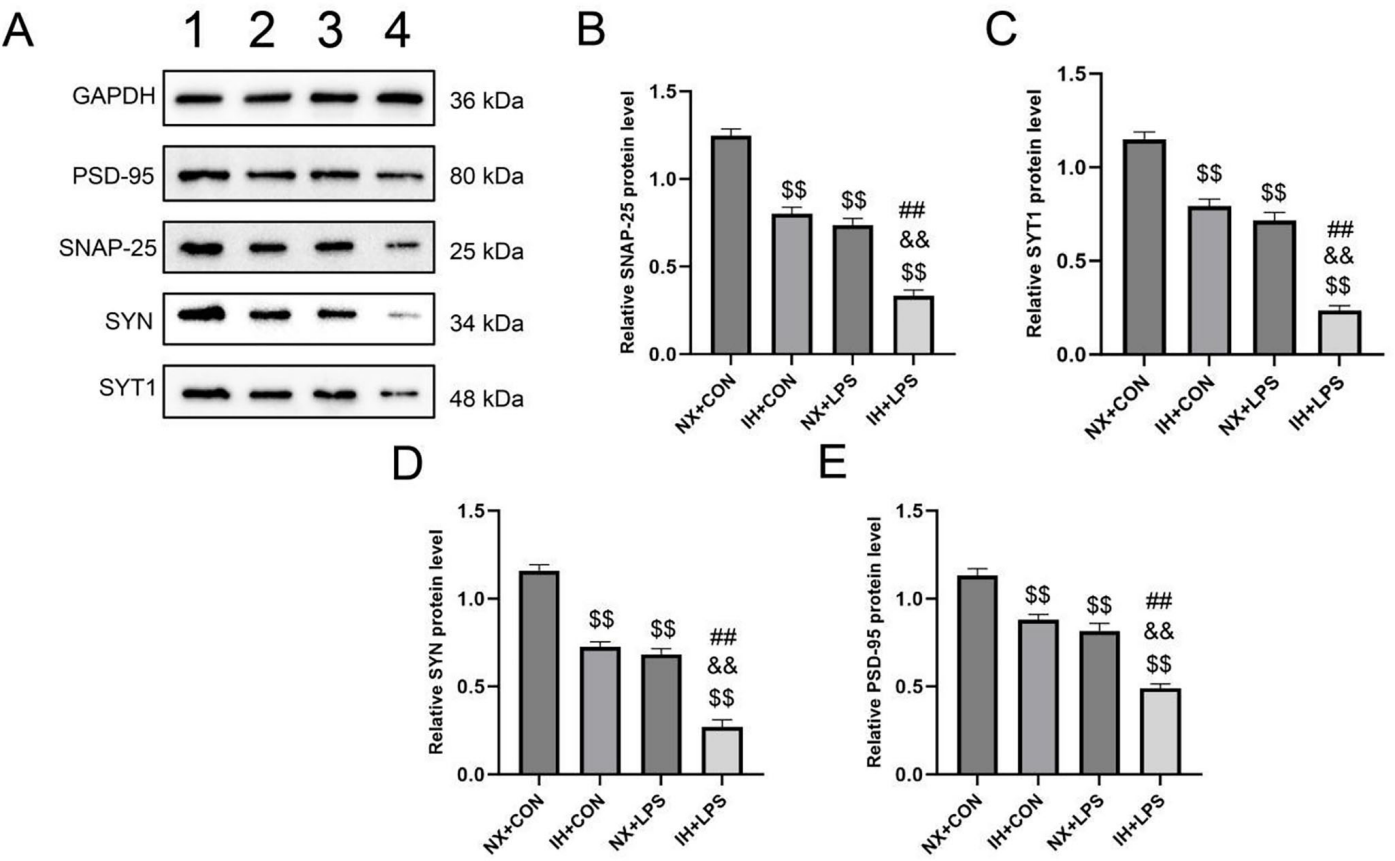
IH further decreased the hippocampus protein concentrations of SNAP‐25, SYT1, SYN, and PSD‐95 in the NX + LPS group (*n* = 6). (A) Western blot: NX + CON group corresponds to Band 1, IH + CON group corresponds to Band 2, NX + LPS group corresponds to Band 3, and IH + LPS group corresponds to Band 4. The protein levels of hippocampal (B) SNAP‐25, (C) SYT1, (D) SYN, and (E) PSD‐95 are shown. *^$$^p* < 0.01 based on intergroup comparison with NX + CON group; *^&&^p* < 0.01 for intergroup comparison with IH + CON group; *^##^p* < 0.01 for intergroup comparison with NX + LPS group.

The protein levels of SNAP‐25 (*F*_(3, 20)_ = 100.0), SYT1 (*F*_(3, 20)_ = 99.87), SYN (*F*_(3, 20)_ = 114.2), and PSD‐95 (*F*_(3, 20)_ = 59.53) varied markedly among the four groups (Figure 7A–E). The levels of SNAP‐25, SYT1, SYN, and PSD‐95 protein were markedly lower in NX + LPS group compared to NX + CON group. Moreover, these proteins exhibited a further significant decrease in IH + LPS relative to NX + LPS group, indicating that IH exacerbated the downregulation induced by LPS exposure.

## 4 Discussion

The onset and progression of neuropsychiatric disorders are often accompanied by neuroinflammation. Chronic IH, as a low‐grade inflammatory stressor, can respond to and amplify the systemic immune response induced by LPS (Zhou et al. 2017). This study demonstrates that chronic IH exacerbates LPS‐induced increases in depression‐like and anxiety‐like behaviors, along with impaired learning and memory functions. The worsening of these abnormal behavioral phenotypes were associated with elevated hippocampal pro‐inflammatory cytokines TNF‐α, IL‐6, and IL‐1β, as well as decreased levels of synaptic‐associated proteins SNAP‐25, SYT1, PSD‐95, and SYN in the hippocampus. These findings provide novel insights into how chronic IH influences LPS‐induced alterations in emotional function and cognitive deficits, offering a fresh perspective on behavioral and brain neurochemical changes under dual strikes of stress and immune activation.

### 4.1 Chronic IH Exacerbates LPS‐Induced Anxiety‐Like and Depression‐Like Behaviors and Cognitive Impairments

Research indicates that sepsis can induce long‐term neurobehavioral changes in humans (Semmler et al. 2013). The establishment of LPS‐induced sepsis models provides efficient and reliable animal systems for infectious disease research. By injecting LPS to mimic bacterial endotoxin‐induced systemic inflammatory responses in rodents, these models can mimic the complex pathophysiological processes of sepsis. LPS has been demonstrated to cause alterations in brain structure and function, leading to emotional dysfunction and cognitive deficits (O'Connor et al. 2009; Zhao et al. 2019). For example, 5 mg/kg LPS induces anxiety‐like and depression‐like behaviors in adult male C57BL/6 mice, as evidenced by elevated plus maze, forced swim test, tail suspension test, and sucrose preference tasks (Anderson et al. 2015). Note that 0.83 mg/kg LPS‐induced cognitive dysfunction in C57BL/6J mice, manifested as reduced preference for unfamiliar objects and locations in the novel object/place recognition test, and significantly decreased time spent in close interaction in the social recognition test (Guo et al. 2023). Consistent with these findings, our study confirms that LPS can induce increased anxiety‐like and depression‐like behaviors in mice during the open field and elevated plus maze tests, as well as impaired spatial learning and memory abilities in the water maze test. Various adverse environmental factors can influence inflammation, thereby altering disease progression (Yaribeygi et al. 2017). Previous studies have demonstrated that repeated social defeat stress exacerbates LPS‐induced anxiety‐like behavior and memory impairment in mice, as measured by the elevated plus maze, novel object recognition and Y‐maze tests (Ajayi et al. 2022). Interestingly, our results show that compared to the NX + LPS group, mice in the IH + LPS group exhibited significantly reduced time spent, distance traveled, and entry frequency in the central area during the open field test, significantly decreased time spent and entry frequency in the open arms in the elevated plus maze, and reduced the distance and time in the target quadrant in the memory phase of the water maze test. These findings indicate that IH exacerbates LPS‐induced adverse neurobehavioral changes. Consistent with these, studies have shown that adult male C57BL/6 mice exposed to both acute hypobaric hypoxia and LPS took significantly longer time to find the hidden platform during the learning phase than controls, and fewer the number of platform crossings, path lengths, and time spent in the target quadrant during the memory phase in Morris water maze test. These results suggest that mice exposed to both acute hypobaric hypoxia and LPS exhibit cognitive impairment. However, administration of LPS or acute hypobaric hypoxia alone did not induce changes in cognitive behavior (Zhou et al. 2017). These heterogeneous results may stem from variations in the dose and timing of LPS administration, as well as differences between acute hypobaric hypoxia and IH models. Current research indicates that IH for 8 h daily can cause significant brain damage and cognitive decline in adult mice (Wang et al. 2023). However, another study indicated that IH alone did not cause brain damage in newborn rats, but did induce brain damage when simultaneously exposed to LPS (Yang et al. 2005). This suggests that IH in this experiment amplified the adverse effects of LPS rather than merely cumulative effects.

### 4.2 Chronic IH Exacerbates LPS‐Induced Neuroinflammation

It is known that systemic administration of LPS can trigger innate immune responses, which not only activate monocytes/macrophages in peripheral tissues via Toll‐like receptors but also activate microglia in the brain through the BBB, thereby inducing systemic inflammation and neuroinflammation. In the central nervous system, LPS primarily binds to TLR4 on glial cell surfaces, initiating MyD88‐dependent inflammatory signaling pathways that promote the release of pro‐inflammatory cytokines (TNF‐α, IL‐1β, IL‐6), chemokines (CCL2, CXCL10), and inflammatory mediators (nitric oxide, prostaglandin E2). Simultaneously, LPS can also induce microglia/macrophage pyroptosis, further exacerbating inflammation by releasing inflammasomes (Yang et al. 2021). Consistent with this, our results indicate that LPS intraperitoneal injection leads to increased levels of the hippocampal pro‐inflammatory cytokines IL‐1, IL‐6, and TNF‐α, as well as an increase in Iba1 and GFAP positive cells in mice, suggesting LPS can induce hippocampal neuroinflammation. A growing body of research indicates that IH can influence the microenvironment of central nervous system. Chronic IH drives microglia to shift from an anti‐inflammatory M2 phenotype to a pro‐inflammatory M1 phenotype, which activates TLR signaling pathways and increases the release of inflammatory factors (Wang et al. 2021). Hypoxia can also suppress the expression of tight junction proteins in vascular endothelial cells, increasing BBB permeability and allowing inflammatory cells and cytokines from the peripheral circulation to enter the central nervous system, thereby further amplifying the inflammatory response. This study demonstrates that chronic IH elevates levels of pro‐inflammatory cytokines in the hippocampus, indicating that IH can induce neuroinflammation in the hippocampus. Interestingly, IH exacerbates LPS‐induced neuroinflammation, as evidenced by significantly higher TNF‐α, IL‐1, and IL‐6 levels in the IH + LPS group compared to the LPS‐only group. Potential mechanisms by which chronic IH intensifies LPS‐induced neuroinflammation include: (1) Hypoxia directly contributes to microglial activation. It has been reported that oxidative stress generated in hypoxic environments contributes to STAT1 activation in microglia, thereby mediating the pro‐inflammatory M1 phenotype in BV2 cells (Butturini et al. 2019). Hypoxia can significantly activate microglia through TLR4 signaling to activate the NF‐κB pathway, which is considered to play a crucial role in the pathogenesis of neurodegenerative diseases such as Alzheimer's disease (Zhang et al. 2017). It is well established that chronic activation of microglia and the release of pro‐inflammatory cytokines lead to a chronic inflammatory state, resulting in neuronal injury and neurodegeneration. (2) Hypoxia‐inducible factor‐1α (HIF‐1α) serves as a central regulatory molecule for cellular perception of hypoxia signals. Studies indicate that hypoxia can upregulate phosphofructokinase/fructose‐2,6‐bisphosphatase 2 (PFKFB2) transcription. During LPS‐induced acute lung injury, the HIF‐1α‐PFKFB2 axis may exacerbate inflammation by promoting dendritic cell maturation through glycolytic reprogramming (Yuan et al. 2025). (3) IH can increase BBB permeability (Roche et al. 2024; Lim and Pack 2014), potentially enhancing the efficiency of peripheral inflammatory factors entering the brain. From a neurobehavioral perspective, the elevated hippocampal pro‐inflammatory cytokines levels driven by IH and LPS play a causal role in mediating the observed affective deficits. Overexpressed IL‐1β can directly act on neural progenitor cells to suppress adult hippocampal neurogenesis, which is a major cellular substrate underlying depressive‐like behaviors (Koo and Duman 2008). Furthermore, these pro‐inflammatory cytokines downregulate brain‐derived neurotrophic factor expression and disrupt monoaminergic neurotransmission—such as activating indoleamine 2,3‐dioxygenase to deplete central serotonin levels—thereby destabilizing downstream emotional regulation networks and ultimately culminating in the observed anxiety‐ and depressive‐like behavioral phenotypes (Sublette and Postolache 2012; Gan and Lee 2025).

### 4.3 Chronic IH Exacerbates LPS‐Induced Synaptic Damage

Synaptic plasticity is considered the biological basis for emotional and cognitive processes (Zhang et al. 2021; Cha et al. 2016). Numerous preclinical experiments revealed that impaired synaptic plasticity is the primary cause of neurobehavioral abnormalities induced by various adverse external factors. For instance, studies indicate that rats exposed to bisphenol A exhibit reduced CA1 and DG dendritic spine densities and decreased DG dendritic complexity in the hippocampus, and lead to impaired learning and memory functions (Zhang et al. 2019). Recent studies also indicate that LPS‐induced neuroinflammation is accompanied by altered synaptic patterns, manifested as reduced excitatory synaptic transmission in hippocampal CA1 during early inflammatory stages followed by elevated inhibitory synaptic transmission. Both of these abnormalities impair hippocampal LTP and ultimately result in cognitive dysfunction (Wu et al. 2025). SNAP‐25, SYT1, PSD‐95, and SYN are synapse‐associated proteins closely related to synapses. Researches have demonstrated that LPS can inhibit the synthesis of synapse‐associated proteins through related inflammatory pathways (Sun et al. 2025). Consistent with this, the current results demonstrated that LPS can lead to reduced mRNA and protein levels of hippocampal SNAP‐25, SYT1, PSD‐95, and SYN compared to the control group. These findings indicate that LPS causes decreased transcription and translation levels of hippocampal synapse‐associated proteins SNAP‐25, SYT1, SYN and PSD‐95 in the hippocampus. Notably, compared to the NX + LPS group, the IH + LPS group exhibited further reductions in hippocampal SNAP‐25, SYT1, SYN, and PSD‐95 mRNA and protein levels, potentially attributable to the more intense inflammatory response induced by chronic IH. Previous studies also support this finding, indicating that the mechanism by which chronic sleep deprivation exacerbates LPS‐induced neurobehavioral abnormalities involves synaptic defects, manifested as decreased levels of synaptic proteins BDNF, SYN, and PSD‐95.

Mechanistically, these key synaptic proteins are essential for maintaining the structural stability of dendritic spines, synaptic vesicle docking, and the anchoring of neurotransmitter receptors; therefore, their downregulation directly impairs synaptic transmission efficiency and neural connectivity (Duman et al. 2016). Crucially, the hippocampus—particularly its ventral region—serves as a key hub in the classical emotional circuit, regulating anxiety through direct monosynaptic projections to emotional regulation centers such as the medial prefrontal cortex and the basolateral amygdala. The severe depletion of SYN, SYT1, SNAP‐25, and PSD‐95 observed in our study likely impairs synaptic strength and disrupts the balance of projections from the hippocampus to the medial prefrontal cortex and amygdala, leading to a loss of control over emotional responses and ultimately manifesting as the observed depression‐ and anxiety‐like behaviors (Cunniff et al. 2020). Previous studies have also confirmed this conclusion, indicating that the mechanism underlying LPS‐induced neurobehavioral abnormalities following chronic sleep deprivation involves synaptic defects, specifically manifested as reduced levels of the synaptic proteins brain‑derived neurotrophic factor, SYN, and PSD‐95 (Zhang et al. 2024).

This study has the following limitations: (1) We only examined inflammatory factor levels in the hippocampal region and did not detect changes in inflammatory factors or synapse‐related proteins in other brain areas associated with mood and cognition, such as the amygdala and prefrontal cortex. (2) The specific targets by which chronic IH modulates inflammation to amplify LPS‐induced neurobehavioral abnormalities remain unclear. (3) Pharmacological methods were not used to inhibit neuroinflammation and reverse the emotional abnormalities and cognitive impairments induced by IH and LPS. (4) We only assessed behavioral changes in male mice under dual stress conditions and did not explore gender factors. (5) The experiment lacked immunofluorescence or immunohistochemical techniques to localize changes in synapse‐associated proteins within the hippocampus.

## 5 Conclusion

In summary, this study demonstrates that chronic IH exacerbates LPS‐induced cognitive impairments, and depression‐like and anxiety‐like behaviors in mice. The mechanisms underlying these intensified neurobehavioral abnormalities may involve increased pro‐inflammatory cytokines and altered synaptic proteins induced by chronic IH. Therefore, this suggests that mood and cognitive function in patients with OSA deserve attention, and the therapeutic potential of targeting inflammation for intervention is well worth exploring.

## Author Contributions

Y.‐Z.D designed the study, performed the behavioral tests, and drafted the manuscript. Z.‐F.W was responsible for the western blotting, RT‐qPCR, and enzyme‐linked immunoassay. C.D. analyzed the data and constructed the graphs. Y.‐Z.Y. revised the manuscript and was responsible for the completeness and accuracy of the data. All authors read and approved the final manuscript.

## Funding

This work was supported by Young Investigator Cultivation Fund of the First Affiliated Hospital of Anhui Medical University (No. 2020kj01) and Anhui Provincial Health and Medical Research Project (No. AHWJ2025BAd20006).

## Ethics Statement

All animal experiments were reviewed and approved by the Association of Laboratory Animal Sciences and the Center for Laboratory Animal Sciences of the Anhui Medical University (NO.LLSC20190710).

## Conflicts of Interest

The authors declare no conflicts of interest.

## Data Availability

The raw data supporting the conclusions of this article will be made available by the authors, without undue reservation.
